# Otolith radiocarbon signatures provide distinct migration history of walleye pollock around Hokkaido, Japan in the North‐Western Pacific

**DOI:** 10.1002/ece3.11288

**Published:** 2024-06-29

**Authors:** Kozue Ando, Yusuke Yokoyama, Yosuke Miyairi, Osamu Sakai, Tomonori Hamatsu, Yuuho Yamashita, Masayuki Chimura, Toshi Nagata

**Affiliations:** ^1^ Atmosphere and Ocean Research Institute The University of Tokyo Kashiwa Japan; ^2^ Department of Earth and Planetary Science The University of Tokyo Hongo Japan; ^3^ Graduate Program on Environmental Sciences The University of Tokyo Komaba Japan; ^4^ Department of Biogeochemistry Japan Agency for Marine‐Earth Science and Technology Yokosuka Japan; ^5^ Research School of Physics The Australian National University Canberra Australian Capital Territory Australia; ^6^ Fisheries Resources Institute (Kushiro) Japan Fisheries Research and Education Agency Kushiro Japan; ^7^ Fisheries Resources Institute (Yokohama) Japan Fisheries Research and Education Agency Yokohama Japan

**Keywords:** oceanodromous migration, otolith, radiocarbon, radioisotope, walleye pollock

## Abstract

Trace elements and stable isotope ratios in otoliths have been used as proxies for the migration history of teleosts; however, their application in oceanic fishes remains limited. This study reports the first use of radiocarbons in otoliths to evaluate the horizontal migration histories of an oceanic fish species, the walleye pollock *Gadus chalcogrammus*. We conducted radiocarbon analyses of three stocks sourced from Hokkaido, Japan. The radiocarbon concentrations from the outermost portion of the otoliths from the Japanese Pacific, Northern Japan Sea (JS), and Southern Okhotsk Sea (OS) stocks were in general agreement with the seawater radiocarbon concentration of the sampling region, suggesting that pollock of all three stocks generally inhabited the within the sea region where each pollocks were sampled throughout their life cycle. However, the radiocarbon signals also provided some indications that some JS and OS stocks may be migrating between different sea regions. The proposed novel approach of reconstructing the individual migration history of marine fish using radiocarbon in otoliths may help examine fish migration with a higher temporal and spatial resolution that could not be achieved by trace elements and stable isotope ratios.

## Graphical abstract

We analyzed radiocarbon concentration in otoliths of walleye poll.

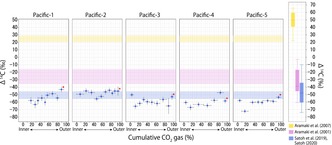

## 1 INTRODUCTION

Accurate and reliable stock identification data are essential for successful fishery management. Although each population group should be managed separately for an optimized outcome (e.g., Begg et al., 1999), the mixing of stocks owing to the ontogenetic migration of species frequently confounds the stock structure. Understanding the migratory history of each stock is beneficial for natural resource conservation. Previous studies have used trace elements and stable isotope ratios of fish tissues as “natural tags” to trace fish migration (Kubota et al., 2015; Tzadik et al., 2017; West et al., 2006). In particular, metabolically inert calcified structures, the otolith, have been widely studied (Amekawa et al., 2016; Campana, 1999; Sturrock et al., 2012). Their chemical composition predominantly reflects that of ambient water at the time of calcification (Campana et al., 1994). Unlike other calcified structures such as endoskeletons or fin rays (Campana et al., 2000; Tzadik et al., 2017), otoliths are not resorbed after formation, and can thus be used as a permanent fingerprint of a fish's life history. Otoliths are composed of >95% calcium carbonate (Campana, 1999), with protein matrices making up the remaining mass. The major elements in otoliths are therefore calcium, carbon, and oxygen, with 47 other minor or trace elements incorporated in the form of proteins or calcium carbonate lattices. Despite the diversity of minor and trace elements in the fish otoliths, most elements and stable isotopes have limited potential as migration proxies for oceanic fish. The horizontal gradients of these chemical properties are generally small or negligible in oceanic waters, except in nearshore regions, which are influenced by terrestrial sources (Kubota et al., 2015; Yokouchi et al., 2017). It is analytically challenging to resolve the small chemical signals of migration in the otoliths of oceanic fish (Proctor et al., 1995; Sturrock et al., 2012; Yokouchi et al., 2017). Furthermore, owing to different physiological processes, most elements show interspecific variability in relationships between otolith compositions and ambient water element concentrations (Brown & Severin, 2009; Rooker et al., 2004). Physiological effects, such as age, somatic growth rate, and gonadal maturation, also affect elemental concentrations in otoliths, which further confounds elemental composition (Kalish, 1991; Sturrock et al., 2014). Therefore, it is necessary to identify a proxy that exhibits high spatial heterogeneity and is unaffected by biological fractionation.

Recently, radiocarbon (^14^C) has emerged as a potentially useful tracer for examining the migration and feeding history of marine fish and mammals (Eisenmann et al., 2017; Larsen et al., 2018; Miyairi et al., 2023). In otoliths, radiocarbon is used to confirm the age of fish (Andrews et al., 2011; Campana, 1997; Kalish et al., 1996). As otolith carbon is largely derived from ambient water (Solomon et al., 2006), it is possible to derive the year of birth of fish by comparing the radiocarbon of the otolith core with that of seawater. One advantage of using ^14^C as an ecological tracer is that ^14^C content is reported as Δ^14^C corrected for isotope fractionation (Stuiver & Polach, 1977). Unlike conventional tracers, Δ^14^C is a proxy that is purely dependent on its source (for the calcium carbonate in otoliths, mainly dissolved inorganic carbon (DIC) in seawater), without the confounding effects of animal physiology and isotope fractionation (Larsen et al., 2018). The large geographic gradient of Δ^14^C values of DIC in the upper oceans constitutes another substantial advantage for ^14^C as a tracer (Lan et al., 2023; Servettaz et al., 2019). ^14^C produced in the upper atmosphere enters the ocean through the air–sea gas exchange of carbon dioxide and is transferred to deep waters by global ocean circulation (McNichol & Aluwihare, 2007). Because it is subject to radioactive decay (half‐life of 5730 years), ^14^C is depleted in deep waters that have been isolated from the air–sea gas exchange for decades or centuries. In low‐ to middle‐latitude oceans where the thermocline is steep, surface waters are isolated from ^14^C depleted subsurface waters and display high Δ^14^C values. In contrast, in upwelling regions, the intrusion of old water from deep layers results in a decrease in Δ^14^C values in the upper layers (Toggweiler et al., 2019). Nuclear weapons testing in the 1950s and 1960s, which rapidly increased atmospheric and surface ocean Δ^14^C values, magnified the gradient of Δ^14^C in the upper ocean (Kumamoto et al., 2013; McNichol & Aluwihare, 2007). Although this artificially introduced Δ^14^C has been decreasing due to absorption by atmosphere–ocean mixing, significant “bomb‐peak” signatures remain in upper oceans in the present time. Currently, in the western North Pacific, the western boundary current (Kuroshio current) that originates from the low‐latitude western Pacific is enriched in ^14^C (ca. 50‰: Ishikawa et al., 2021; Yokoyama et al., 2022). In contrast, the subarctic current (Oyashio current) delivers water with low Δ^14^C values of ca. −50‰ (Ishikawa et al., 2021; Ota et al., 2021; Satoh et al., 2019). Therefore, a large geographic gradient of Δ^14^C (−50 to 50‰) appears in oceanic regions influenced by these two current systems. These regions include those around Hokkaido, where a large amount of fishing activity takes place. Similarly, geographic Δ^14^C gradients appear in other oceanic regions influenced by upwelling (e.g., off California and the Southern Ocean; Druffel & Williams, 1991; Toggweiler et al., 2019). Although a strong geographic gradient of Δ^14^C can serve as a potentially powerful marker to examine the horizontal migration of marine organisms (Eisenmann et al., 2017), no previous studies, to the best of our knowledge, have explored the use of otolith radiocarbon to investigate the horizontal migration of fish.

The purpose of this study was to explore the potential application of otolith radiocarbon to reconstruct the migration history of marine organisms using walleye pollock (*Gadus chalcogrammus*), hereafter referred to as pollock, as a model. Pollock is a key species in the North Pacific ecosystem (Bailey et al., 1999) and an important fishery resource (Springer, 1992). It forms meta‐populations around Hokkaido, separating in four fishery stocks: the Japanese Pacific (JP), the Northern Japan Sea (JS), the Southern Okhotsk Sea (OS), and the Nemuro Strait stocks (Mori & Hiyama, 2014; Watanobe, 2008). Previous work has found that the annual stock distribution of pollock changes dramatically depending on environmental factors such as temperature and food availability (Maeda et al., 1993). Early tagging survey results suggest that some exchanges of individuals take place across different regions of Hokkaido (Nishimura et al., 2002; Yoshida, 1982). However, the ontogenetic migration histories of these individuals remain unclear. Understanding the migration patterns of each stock in Hokkaido is essential for supporting appropriate fishery management.

## 2 MATERIALS AND METHODS

### 2.1 Sample collection

**FIGURE 1:**
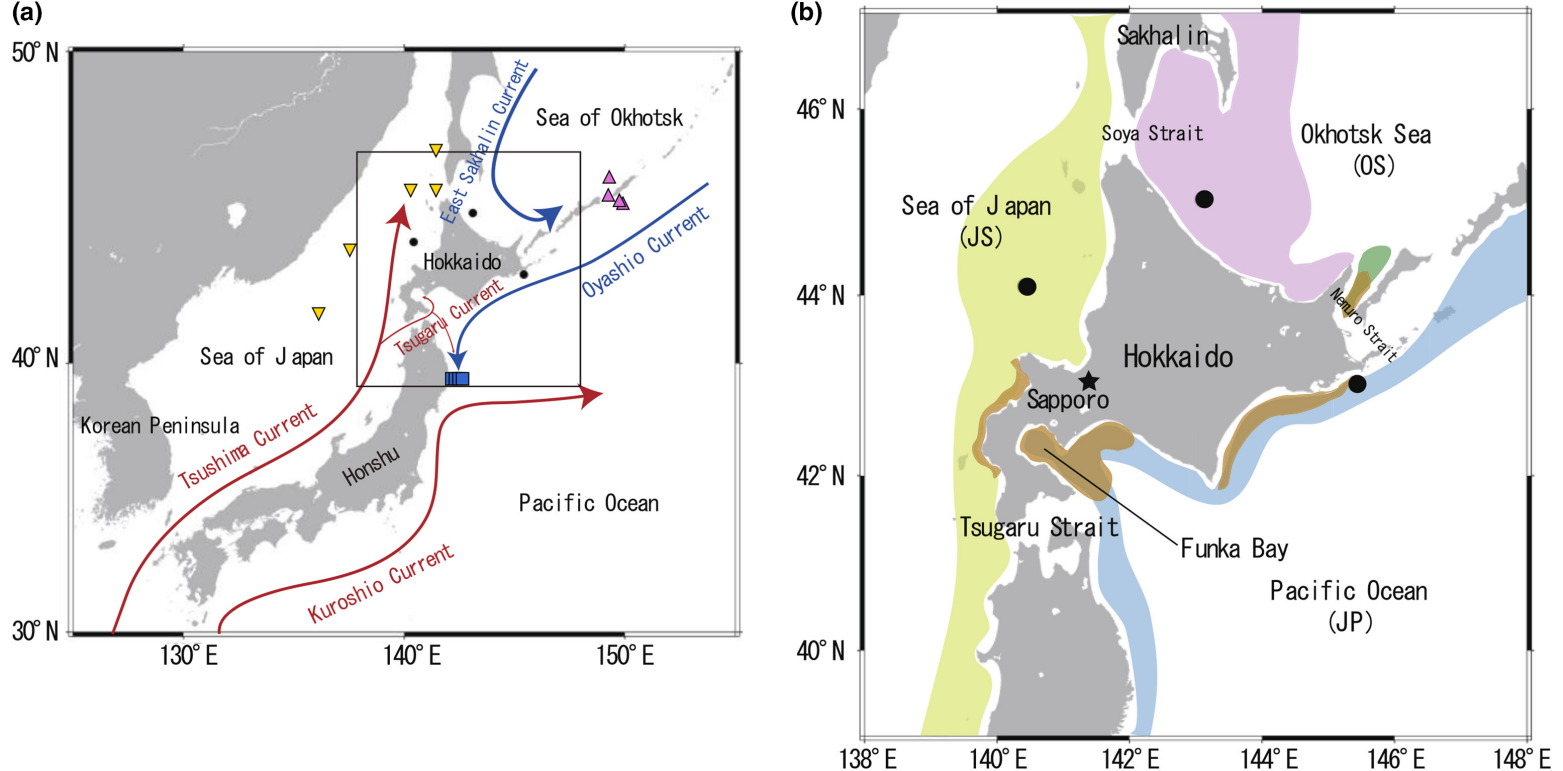
Maps of oceanic region around the study site. (a) General map showing cold, subarctic currents (blue arrows) and warm currents originating from the subtropical region (red arrows) around Japan. Black circles indicate pollock sampling sites. Yellow inverted triangles, pink triangles, and blue squares represent the seawater sampling sites of Aramaki et al. (2007), Aramaki et al. (2001) and Satoh et al. (2019), Satoh (2020), respectively. (b) Close up map of the study area. Black circles indicate the pollock sampling sites. Yellow, blue, and pink shades denote the major distribution areas of JS, JP, and OS stocks, respectively.

Pollock were collected using mid‐water/bottom trawls (RV Kaiyo‐Maru No. 5 and RV Hokko‐Maru cruises) from three regions (JP, JS, and OS) around Hokkaido (Figure 1b, Table A1). After measuring the body length (BL; length from the tip of the lower jaw to the base of the caudal fin) and weight (BW) of the specimens, pairs of sagittal otoliths were removed. Otoliths removed from the large (>400 mm BL) size group, corresponding to an approximately 5‐year‐old or older age class (Hamatsu et al., 2004; Kooka, 2012), were used for radiocarbon analyses (Table 1). Pollock collected from the JP (*n* = 5), JS (*n* = 5), and OS (*n* = 4) regions were named as Pacific‐1 to 5, Japan‐1 to 5, and Okhotsk‐1 to 4, respectively.

**TABLE 1:** Sampling information of walleye pollock analyzed in this study.

Otolith serial number	Region	Sampling date	Sex	Fork length (mm)	Body length (mm)	Body weight (g)	Otolith weight(mg)
23	JS	5/15/2016	F	460	425	606	466.29
25	JS	5/15/2016	F	469	433	562	376.17
26	JS	5/15/2016	M	454	420	537	369.35
28	JS	5/15/2016	F	454	420	537	390.34
29	JS	5/15/2016	M	445	409	536	304.00
53	JP	7/4/2015	M	484	448	611	367.27
54	JP	7/4/2015	M	446	412	651	362.18
56	JP	7/4/2015	F	468	438	705	470.38
59	JP	7/4/2015	F	421	393	499	290.07
51	JP	7/4/2015	M	442	411	567	344.35
83	OS	4/20/2016	F	438	406	587	267.72
84	OS	4/20/2016	M	432	398	608	251.82
86	OS	4/20/2016	M	474	439	924	301.82
82	OS	4/20/2016	F	456	421	611	384.54

*Note*: In the sex column, “F” represents female and “M” represents male pollock. Details of trawl locations are shown in Table A1.

### 2.2 Sample preparation and graphitization

We analyzed 14 otolith samples (five each from JS and JP and four from OS) (Table 1). Otoliths were weighed and reacted with 85% H_3_PO_4_ (80°C) to collect CO_2_ gas using a stepwise dissolution procedure (Burr et al., 1992; Miyairi et al., 2023; Yokoyama et al., 2000). Eight to 10 discrete gas samples were collected from each otolith before dissolving the entire otolith. The reaction time for each gas collection time was determined by the otolith weight so that an approximately equal amount of CO_2_ gas (3–6 mg C depending on the size of the otolith) was collected at each step. Then the CO_2_ gas was cryogenically trapped in a vacuum line, reduced to solid graphite (Yokoyama et al., 2022), and analyzed using a single‐stage accelerator mass spectrometer at the Atmosphere and Ocean Research Institute at The University of Tokyo, Japan (Yokoyama et al., 2019). Radiocarbon values were reported as Δ^14^C using Equation (1) (Stuiver & Polach, 1977):

(1) $${∆}^{14}C\;\left(‰\right)=\delta^{14}C-2\frac{\delta^{13}C+25}{1+\frac{\delta^{14}C}{1000}}$$

where δ^13^C and δ^14^C are defined as the per mil (‰) deviation from the standard (Vienna Pee Dee Belemnite for δ^13^C, oxalic acid for δ^14^C).

The carbon obtained using the stepwise acid dissolution method was derived solely from calcium carbonate. As phosphoric acid reacts only with calcium carbonate, the remaining protein is preserved in the phosphoric acid solution. This method is typically used in carbonate radiocarbon analysis including that of otoliths (e.g., Burr et al., 1992; Grammer et al., 2015; Miyairi et al., 2023; Yokoyama et al., 2000).

The stepwise dissolution procedure of otoliths (Miyairi et al., 2023) yields a series of discrete gas collections from the outer portion (1st sample collection) to the inner core (*n*th sample collection) (Table A2). To evaluate the life stage represented by each gas sample, we calculated the cumulative amount of gas normalized by total gas collected in inverse order of gas sample collection (for the convenience of data presentation) using the following equations:

(2) $$x_{n-\left(k-1\right)}=100\times \left(\frac{\sum_{k=2}^{n}g_{n-\left(k-2\right)}}{\sum_{k=1}^{n}g_{k}}+\frac{g_{n-\left(k-1\right)}}{2\times \sum_{k=1}^{n}g_{k}}\right)\;\text{when}\;k<n$$

(3) $$x_{n-\left(k-1\right)}=100\times \frac{g_{n-\left(k-1\right)}}{2\times \sum_{k=1}^{n}g_{k}}\;\text{when}\;k=n$$

where *x*_*n*–(*k*–1)_ is the Δ^14^C of the *k*th gas collection, *g*_*k*_ is the amount of CO_2_ (hPa) obtained in the *k*th gas collection and *n* is the total number of CO_2_ gas collections from one otolith sample (*n* = 10 for Japan‐1,2,5, Pacific‐1,2,3, and Okhotsk‐1,3,4; *n* = 9 for Japan‐4, Pacific‐4, and Okhotsk‐2; *n* = 8 for Japan‐3 and Pacific‐5), with an error of *x*_*n*_ expressed as ±$\frac{g_{n}}{2}$. This would allow the timing of otolith formation to be estimated. As the gas was not collected in equal amounts, this would provide a more plausible estimate of the timing of otolith formation than plotting individual measurement points at equal intervals. The life stage was scored using *x* values, with the entire lifespan scaled between 0 (innermost core portion) and 100 (outermost edge) %. The plot of Δ^14^C values of discrete gas samples over *x* values (life stage) for each individual was used to infer the time‐course changes of otolith Δ^14^C values during its life (see Section 4 for notes on the uncertainty associated with the time resolution of otolith radiocarbon records).

Mean ranks of otolith Δ^14^C values among individuals belonging to each stock were compared by the Kruskal–Wallis test followed by the Steel–Dwass test using R v.4.3.1 (R Core Team, 2023).

## 3 RESULTS

**FIGURE 2:**
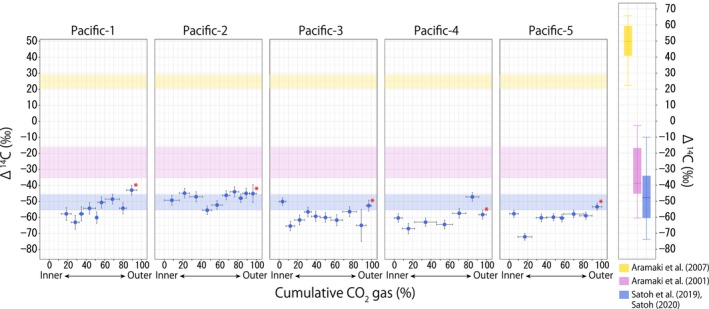
Otolith radiocarbon records of walleye pollocks from the Japanese Pacific region (*n* = 5). *X*‐axis values (cumulative CO_2_ gas collected) indicate the life stage of each pollock, with the entire lifespan scaled between 0 and 100%. Vertical error bars represent the analytical error of the Δ^14^C measurement. Horizontal error bars are calculated as $\frac{g_{n}}{2}$ from Equation (2). Δ^14^C values marked with red asterisk indicate data from the outermost portion of the otolith. Blue shading represents the 95% confidence interval of asterisked Δ^14^C values, regarded as the proxy of seawater Δ^14^C signature to which the pollock were exposed during approximately the last 10% of their lifespan. Yellow and pink shading indicate asterisk Δ^14^C value ranges for the JS stock (Figure 3) and OS stock (Figure 4), respectively. One measurement point from Pacific‐1, two from Pacific‐5, and three from Pacific‐4 were excluded due to sample loss or failure in AMS measurement. Box plot shows the surface (0–200 m) seawater Δ^14^C values from past studies. Sampling sites are shown in Figure 1a.

**FIGURE 3:**
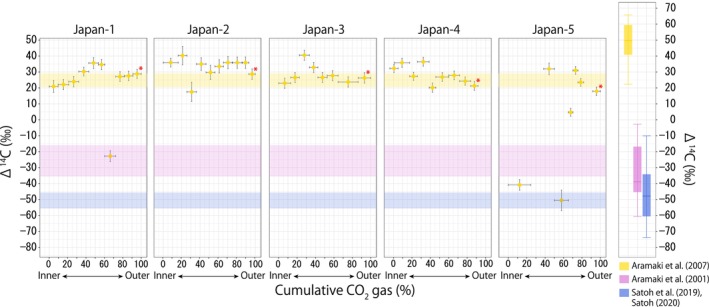
Otolith radiocarbon records of walleye pollock from Sea of Japan region (*n* = 5). Iconography is as in Figure 2. One measurement point from Japan‐1, from Japan‐3, and three from Japan‐5 were excluded due to sample loss or failure in AMS measurement.

The Δ^14^C values of the outermost portion of otoliths were −50.55 ± 4.93, 24.57 ± 3.78, and −25.64 ± 9.78 for the JP, JS, and OS regions, respectively (Table 2). Because the outermost portion of samples generally contributed approximately 10% of the collected CO_2_ gas (except for one individual in the JP stock [Pacific‐3] which contained 5.7% of total gas in the outermost portion), the values represent the mean Δ^14^C value of ambient seawater DIC in which each fish had spent the final 10% of their life history. This is roughly equivalent to 0.5 years if the pollock was 5 years old at the time of sample collection. However, the time resolution of the otolith radiocarbon records should be interpreted with caution (see Discussion). We obtained 8–10 Δ^14^C values for each otolith using a stepwise dissolution method (Table A3), representing a time series across the individual's entire lifetime (the otolith radiocarbon record). The otoliths from the JP stock were strongly depleted in radiocarbon (range, −70‰ to −40‰) throughout their lives (Figure 2), and means differed among the five individuals (Table 3). In contrast, otoliths from the five individuals of the JS stock were enriched with radiocarbon (range, 20‰–40‰), except that some gas samples collected from Japan‐1 and Japan‐5 displayed anomalously low values (−20‰ to −50‰) at specific life stages (Figure 3). Specifically, in Japan‐1, Δ^14^C abruptly decreased from 34.62‰ (at *x* = 56.92%) to −22.76‰ (*x* = 66.32%), followed by a return to the original level of 27.11‰ (*x* = 76.90%). In Japan‐5, Δ^14^C values were anomalously low at life stages of *x* = 12.19% (Δ^14^C, −40.85‰) and *x* = 57.69% (−50.53‰). Mean ranks of Δ^14^C values of otoliths significantly differed among these individuals as well (Table 4).

**TABLE 2:** Mean of the outermost otolith Δ^14^C for the three walleye pollock stocks analyzed in this study.

Sea region	Δ^14^C (‰)	Error (‰)
JP	−50.55	±4.93
JS	24.57	±3.78
OS	−25.64	±9.78

*Note*: Errors are 95% confidence intervals of otolith Δ^14^C used for the calculation. Japanese Pacific (JP): *n* = 5, Sea of Japan (JS): *n* = 5, Southern Okhotsk Sea (OS): *n* = 4.

**TABLE 3:** Mean of the outermost otolith Δ^14^C for Japanese Pacific walleye pollock specimens analyzed in this study.

Specimen	Mean Δ^14^C ± SD (‰)	Steel–Dwass test			
		Pacific‐2	Pacific‐3	Pacific‐4	Pacific‐5
Pacific‐1	−54.46 ± 6.21	NS	NS	NS	NS
Pacific‐2	−47.81 ± 3.69		*p* = .004	*p* = .021	*p* = .005
Pacific‐3	−58.94 ± 4.99			NS	NS
Pacific‐4	−59.73 ± 6.46				NS
Pacific‐5	−60.14 ± 5.37				

Abbreviations: NS, not significant (*p* < .05, Kruskal–Wallis test followed by Steel–Dwass test); SD, standard deviation.

**TABLE 4:** Mean of the outermost otolith Δ^14^C for Sea of Japan walleye pollock specimens analyzed in this study.

Specimen	Mean Δ^14^C ± SD (‰)	Steel–Dwass test			
		Japan‐2	Japan‐3	Japan‐4	Japan‐5
Japan‐1	22.80 ± 16.72	NS	NS	NS	NS
Japan‐2	32.80 ± 6.31		NS	NS	p = .04
Japan‐3	28.37 ± 5.73			NS	NS
Japan‐4	28.00 ± 5.83				NS
Japan‐5	2.52 ± 34.28				

Abbreviations: NS, not significant (*p* < .05, Kruskal–Wallis test followed by Steel–Dwass test); SD, standard deviation.

**FIGURE 4:**
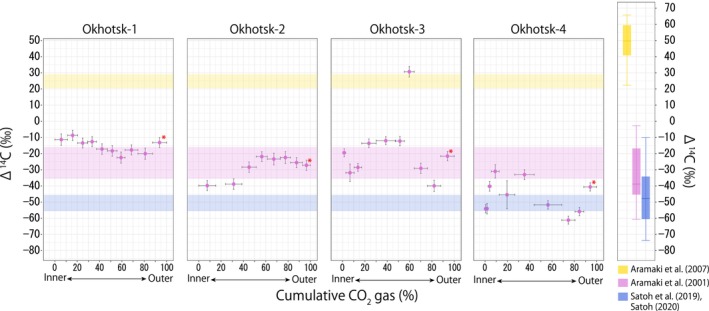
Otolith radiocarbon records of walleye pollock from Southern Okhotsk Sea region (*n* = 4). Iconography is as in Figure 2. Two measurement points from Okhotsk‐3 were excluded due to failure in AMS measurement.

The patterns in the otolith radiocarbon records for the four individuals collected from the OS region were more diverse than those from other regions (Figure 4). While Okhotsk‐1 displayed a relatively stable Δ^14^C record within the range of −8‰ to −23‰, Okhotsk‐2 showed an increasing trend from about −40‰ (at *x* = 7.60‰–31.31%) to about −20‰ (at *x* = 56.50‰–77.63%). In Okhotsk‐3, Δ^14^C value abruptly increased from −12.24‰ (*x* = 51.34%) to 30.66‰ (*x* = 59.76%) and then decreased to −29.10‰ (*x* = 70.11%). Δ^14^C values of Okhotsk‐4 were variable and tended to be lower (range, −31‰ to −61‰) than other individuals. Mean ranks of Δ^14^C values again significantly differed among individuals (Table 5).

**TABLE 5:** Mean of the outermost otolith Δ^14^C for Okhotsk Sea walleye pollock specimens analyzed in this study.

Specimen	Mean Δ^14^C ± SD (‰)	Steel–Dwass test		
		Okhotsk‐2	Okhotsk‐3	Okhotsk‐4
Okhotsk‐1	−15.50 ± 4.30	*p* = .003	NS	*p* = .001
Okhotsk‐2	−27.44 ± 7.28		NS	NS
Okhotsk‐3	−17.77 ± 19.37			*p* = .002
Okhotsk‐4	−46.73 ± 10.23			

Abbreviations: NS, not significant (*p* < .05, Kruskal–Wallis test followed by the Steel–Dwass test); SD, standard deviation.

## 4 DISCUSSION

### 4.1 Radiocarbon content in the outermost portion of otolith agreed with regional seawater radiocarbon signature

The Δ^14^C values of the outermost portion of otolith were distinct among pollock stocks, displaying low, intermediate, and high values for JP, OS, and JS stocks, respectively (Figures 2, 3, 4, Table 2). These values are generally consistent with seawater Δ^14^C values previously reported in the corresponding regions or water masses (Aramaki et al., 2001, 2007; Satoh, 2020; Satoh et al., 2019). Previous work has found that in the Oyashio‐influenced region (the JP region), the median Δ^14^C value (interquartile range) was −42‰ (−34 to −60) [estimated from the data reported by Satoh et al. (2019) and Satoh (2020) for the depth of 0–200 m], close to the range of Δ^14^C values for the otolith outermost portion of the JP stock (c. 2). Aramaki et al. (2007) investigated the Δ^14^C values of seawater in the Sea of Japan (the JS region), reporting that seawater Δ^14^C ranged from 20% to 70‰ in the upper layer. Although the upper range of these measurements exceeded the range we observed in the outermost otolith portion of the JS stock (Figure 3), this could be due to the timing of sample collection. As Aramaki et al.'s samples were collected in 1998, Δ^14^C values were higher than at present due to the more substantial effects of bomb carbon in water masses originating from the subtropical gyre (Kumamoto et al., 2013). In the Okhotsk Sea (the OS region), a study conducted in the Kuril Islands found that seawater Δ^14^C values mostly ranged from −60% to 0‰ in the upper layer (0–200 m) (Aramaki et al., 2001). These values are intermediate to those reported for the Oyashio current (Satoh, 2020; Satoh et al., 2019) and the Sea of Japan (Aramaki et al., 2007), roughly corresponding to the range of otolith outermost portion Δ^14^C values of the OS stock (Figure 4). Although a rigorous comparison would require the simultaneous sampling of pollock and seawater, the available data suggest that the radiocarbon content of the outermost otolith portion agreed reasonably well with ambient seawater radiocarbon signatures.

### 4.2 Time resolution of each step and its associated uncertainties

Using a stepwise dissolution approach, we successfully obtained time‐series records of otolith radiocarbon. Assuming that each gas sample corresponded to an equal length of otolith lifespan, the time resolution of our chronological reconstruction was 0.5–0.6 years (i.e., the age of fish (5 years) divided by eight, nine, or 10). Note that the time resolution range was an approximate value. Otolith growth may decrease with fish age due to declining somatic growth (Hanson & Stafford, 2017), implying that gases collected from the outer portion of the otolith have a lower time resolution (that is, integrate information over a longer period) than those collected from the inner core. The complex morphology of otoliths adds another uncertainty to the estimation of the time resolution, as the growth rate of otoliths can differ depending on their axes (Galley et al., 2006; Gauldie & Nelson, 1990). Future studies should examine the relationship between radiocarbon signatures and pollock growth estimated from otolith readings with the aid of the otolith micro‐milling technique (Høie et al., 2004).

### 4.3 Resolving fish migration history: Did pollock move to different regions?

Five individuals from the JP stock displayed stable radiocarbon values, indicating that they remained in the JP region throughout their lives. Our findings are consistent with the results of previous studies suggesting that pollock have several spawning grounds in the JP region, displaying dispersed feeding and homing migration within the region (Figure 1, Nishimura et al., 2002). Interestingly, mean ranks of Δ^14^C values differed significantly among JP stock individuals (Table 3), likely reflecting differences in the location of spawning ground, migration trajectory, or both, within the JP region. Future studies examining the fine‐scale distribution patterns of seawater radiocarbon in the JP region would be helpful in resolving the differentiation in migratory behavior within the region among JP individuals.

The otolith radiocarbon data for the JS stock individuals provided distinct signatures, suggesting that the two individuals from this stock migrated across regions (Figure 3). While three individuals (Japan‐2,3,4) with stably high Δ^14^C values likely remained in the JS region throughout their lives, anomalously low Δ^14^C values recorded for Japan‐1 and Japan‐5 indicate that they were exposed to radiocarbon‐depleted seawater at certain stages of their lives (Figure 3). For Japan‐1, the decrease of Δ^14^C from 34.62‰ to −22.77‰ at *x* = 66.32%, followed by a return to the original level of 27.11‰, indicates that this individual moved temporarily from JS to probably the OS region during its adult stage. Similarly, Japan‐5 appears to have stayed in a Δ^14^C‐depleted region (likely JP) when it was young (*x* = 12.19%), migrated to the JS region after growing to maturity (*x* = 44.25%), re‐visited JP at *x* = 57.69%, and then remained in JS for the rest of its life. These inferences on migration destinations are based on the similarity of Δ^14^C values between each otolith section and the regional radiocarbon signature. That is, anomalous Δ^14^C values of −22.77‰ and ca. −40‰ to −50‰ for Japan‐1 and Japan‐5 respectively correspond to the intermediate (OS) and low (JP) Δ^14^C signatures of seawater, respectively (Table 2). However, this destination assignment is subject to uncertainty, especially for Japan‐1, because the radiocarbon signal recorded in the destination region was “diluted” by the Δ^14^C signature in the home region if the staying period in the destination region was less than the time resolution of our record (approximately 6 months). This means that the intermediate Δ^14^C signal in Japan‐1 can be explained by either a short visit to the JP region (low Δ^14^C) or a long visit to the OS region (intermediate Δ^14^C). This should be solved in future studies by improving the temporal resolution of otolith radiocarbon records.

Diverse patterns were found in the otolith radiocarbon records of individuals collected from the OS region, for which two possible explanations exist that are not mutually exclusive. First, the OS stock may consist of individuals with diverse migratory behaviors, including those who tend to stay in the OS region (Okhotsk‐1), those who display temporary migration to the JS region with a high radiocarbon signature (Okhotsk‐3), and those who tend to migrate between the OS and JP regions (Okhotsk‐4). Second, the distribution of seawater radiocarbons in the OS region might be more variable across the season, across localities, or both, than in other regions. For example, the seasonal exchange of seawater between adjacent regions and local upwelling could introduce large spatial and temporal heterogeneities in radiocarbon distributions even at a scale of a few to tens of kilometers, depending on the oceanographic settings (Satoh, 2020). Such heterogeneities in seawater radiocarbon distribution within a region complicate the interpretation of otolith radiocarbon records. Improving our understanding of the temporal and spatial dynamics of radiocarbons in target regions will be a major challenge for future studies.

Our interpretation of otolith radiocarbon records as evidence of the migration of Japan‐1 and Japan‐5 across regions (horizontal migration) may be challenged by the proposition that the otolith radiocarbon record reflects the vertical movement of pollock within the same region given that the radiocarbon content of seawater generally decreases with depth (Larsen et al., 2018; Miyairi et al., 2023). We argue against this proposition based on the known vertical migration pattern of pollock and Δ^14^C pattern observed in the individuals analyzed in our study. Pollock ontogenetically change their distribution depth (Honkalehto et al., 2010). Around Hokkaido, the mean habitat depths (average of night and daytime habitat depth) for juvenile and adult pollock are approximately 50–100 m and 200 m, respectively (Honda et al., 2004; Itaya et al., 2009; Kooka et al., 1998; Miyashita et al., 2004; Shida, 2001). This estimated habitat depth difference between juveniles and adults is equivalent to the depth‐dependent offset of 20%–30‰ Δ^14^C in seawater (Aramaki et al., 2007). Therefore, the ontogenetic shift in habitat depth alone would not account for the large shift (60%–80‰) in otolith Δ^14^C observed in Japan‐1 and ‐5. Furthermore, ontogenetic vertical shifts in fish habitat depth generally occur gradually throughout their life (Honda et al., 2004; Itaya et al., 2009), while the migration pattern inferred from the pollock otolith radiocarbon records was rather abrupt. In summary, a large, abrupt change in otolith Δ^14^C cannot be explained by a known ontogenetic vertical migration pattern of pollock.

## 5 CONCLUSIONS AND FUTURE PERSPECTIVES

Our results corroborate the emerging notion that radiocarbon is a powerful tracer for resolving the migratory behavior of fish (Larsen et al., 2018; Miyairi et al., 2023). The application of the otolith radiocarbon recording approach to pollock around Hokkaido provided new insights into the migration of this enigmatic species across these regions. Most strikingly, the otoliths of some individuals had a record of strong radiocarbon signals that differed from those in their sampling regions, indicating temporary migration to adjacent regions. This finding not only supports previous suggestions based on tagging surveys that there are exchanges of individuals across regional stocks around Hokkaido (Yoshida, 1982) but also provides novel information regarding the migration history of each individual. The number of individuals analyzed for each region (*n* = 4 or 5) in this study was insufficient to assess the proportion of migrant and non‐migrant individuals within each regional stock. Nonetheless, the migration signal detected in two of five (JS stock) and one of four (OS stock) individuals suggests that migration is not rare. Future efforts should focus on increasing the number of individuals analyzed for each stock. In this regard, our stepwise dissolution procedure has an advantage over the micromilling approach in terms of simplicity, cost, and labor intensity (Miyairi et al., 2023). In future studies, there is room for improving the accuracy of time resolution by coherently examining fish age (from otolith readings) and radiocarbon. Furthermore, the use of advanced AMS with an analytical detection limit of 10 μg C per sample promises improvements in the time resolution of the otolith radiocarbon record (Yokoyama et al., 2022). Finally, improving the understanding of radiocarbon distribution patterns at regional and sub‐regional scales with the aid of modeling approaches (St John Glew et al., 2021) is necessary to refine the evaluation of individual migration trajectories using otolith radiocarbon records.

## AUTHOR CONTRIBUTIONS

**Kozue Ando:** Investigation (equal); methodology (lead); writing – original draft (lead); writing – review and editing (lead). **Yusuke Yokoyama:** Conceptualization (lead); investigation (supporting); methodology (supporting); supervision (lead); writing – original draft (supporting); writing – review and editing (supporting). **Yosuke Miyairi:** Investigation (supporting); methodology (supporting); writing – review and editing (supporting). **Osamu Sakai:** Investigation (supporting); methodology (supporting); writing – review and editing (supporting). **Tomonori Hamatsu:** Investigation (supporting); methodology (supporting); writing – review and editing (supporting). **Yuuho Yamashita:** Investigation (supporting); methodology (supporting); writing – review and editing (supporting). **Masayuki Chimura:** Investigation (supporting); methodology (supporting); writing – review and editing (supporting). **Toshi Nagata:** Investigation (supporting); methodology (supporting); writing – review and editing (supporting).

## FUNDING INFORMATION

The YS‐AMS was funded by Funding Program for Next Generation World Leading Researchers (GR031) to YY and JST CREST (JPMJCR13A4; JPMJCR23J6). This work was supported by a grant from the Japan Society for the Promotion of Science (JSPS) KAKENHI (20H00193; 23KK0013). This work was supported in part by the Fisheries Agency of Japan under the project of ‘Assessment of Fisheries Stocks in the Waters Around Japan’.

## CONFLICT OF INTEREST STATEMENT

All other authors declare they have no competing interests.

## Data Availability

All data are available in the main text or in the Appendix.

## Appendix

**TABLE A1:** Details of trawl locations and date.

Region	Cruise Ship	Sample collection date	Trawl location number	Latitude (°N)	Longitude (°E)	Trawl depth (m)	Trawl net temperature (°C)	Number of walleye pollocks captured
JS	Hokko Maru	2016/5/15	MT‐5	44°09′9276	140°43′2586	407	0.9	146
JP	Daigo Kaiyo Maru	2015/7/4	MT‐34	43°03′190	145°41′975	99	3.8	845
OS	Daigo Kaiyo Maru	2016/4/20	A4	45°03′8	143°11′5	134	4.6	223

**TABLE A2:** Gas collection rate of otoliths (*n* = 14) analyzed in this study.

	Otolith serial number	Otolith weight (mg)	Gas collection rate (%)	Total gas collection (hPa)	1st	2nd	3rd	4th	5th	6th	7th	8th	9th	10th
japan‐1	29	304.00	76.62	4472	400	425	416	530	311	465	489	488	516	432
japan‐2	26	369.35	73.48	5211	364	382	550	505	484	504	546	536	495	845
japan‐3	28	390.34	56.44	4230	578	925	500	429	389	462	395	552	N/A	N/A
japan‐4	23	466.29	56.99	5079	407	615	599	710	369	650	430	840	459	N/A
japan‐5	25	376.17	64.16	4634	382	460	285	251	230	705	540	lost	651	1130
pacific‐1	53	367.27	59.31	4182	472	318	641	346	100	547	190	362	454	752
pacific‐2	56	470.38	70.88	6401	470	482	262	616	534	730	641	903	703	1060
pacific‐3	59	290.07	62.70	3492	200	363	526	446	410	329	261	370	336	251
pacific‐4	51	344.35	55.90	3696	300	Lost	522	535	643	880	495	246	75	N/A
pacific‐5	54	362.18	57.59	4005	373	605	450	571	170	884	545	407	N/A	N/A
okhotsk‐1	86	301.82	77.38	4484	560	590	516	323	384	440	355	403	403	510
okhotsk‐2	84	251.82	68.71	3322	233	357	306	384	330	428	488	291	505	N/A
ohkotsk‐3	83	267.72	75.33	3872	467	460	460	342	310	669	502	260	292	110
ohkotsk‐4	82	384.54	64.89	4791	555	374	570	1185	829	672	340	140	82	44

*Note*: The gas collection rate was calculated from the total gas collection divided by the theoretical amount of gas obtained when assuming that CaCO_3_ constitutes 100% of the otolith. “1st” to “10th” refers to the amount of gas collected in the first to tenth gas collection (hPa; measurement tube volume, 11.5 mL; therefore, 160 hPa = 1 mg C). N/A: gas collection was not conducted due to the formation of cracks in the otolith. Graphitized gases that did not produce sufficient amount of beam during the measurement (9th collection in Pacific‐4 and Japan‐5) were excluded in Table A3.

**TABLE A3:** δ^13^C and Δ^14^C values of walleye pollock otolith analyzed in this study.

Lab No.	Sample	δ^13^C (‰)	Error (±1*σ*)	Δ^14^C (‰)	Error (±1*σ*)
YAUT‐058616	suke29‐1	1.53	±1.38	28.77	±2.81
YAUT‐058617	suke29‐2	−1.52	±1.42	27.53	±2.92
YAUT‐058618	suke29‐3	−1.15	±1.68	27.11	±3.09
YAUT‐058619	suke29‐4	0.01	±2.09	−22.77	±3.43
YAUT‐058623	suke29‐5	−0.47	±1.76	34.62	±3.19
YAUT‐058624	suke29‐6	−2.18	±1.96	35.58	±3.42
YAUT‐058625	suke29‐7	−0.66	±1.27	30.23	±2.72
YAUT‐058916	suke29‐8	−1.92	±0.67	23.91	±3.30
YAUT‐058917	suke29‐9	−0.90	±0.29	22.09	±3.14
YAUT‐058918	suke29‐10	−11.69	±1.20	20.92	±3.72
YAUT‐059202	suke26‐1	0.24	±1.16	28.67	±3.31
YAUT‐059203	suke26‐2	−2.89	±1.62	35.81	±3.55
YAUT‐059204	suke26‐3	−1.54	±1.95	35.89	±3.86
YAUT‐059205	suke26‐4	−2.42	±2.23	33.51	±4.15
YAUT‐059206	suke26‐5	11.94	±2.42	29.73	±4.42
YAUT‐059209	suke26‐6	−5.18	±2.05	34.97	±3.97
YAUT‐059211	suke26‐7	−1.23	±3.85	17.51	±6.01
YAUT‐059212	suke26‐8	1.24	±4.57	37.36	±7.03
YAUT‐059213	suke26‐9	0.16	±3.50	40.26	±5.78
YAUT‐059215	suke26‐10	3.29	±0.53	35.87	±2.77
YAUT‐059502	suke28‐1	−2.55	±0.65	26.25	±3.28
YAUT‐059503	suke28‐2	−3.53	±0.50	23.71	±3.21
YAUT‐059504	suke28‐3	−2.85	±0.14	27.68	±3.13
YAUT‐059505	suke28‐4	−3.35	±0.24	26.53	±3.14
YAUT‐059506	suke28‐5	−1.68	±0.38	32.84	±3.19
YAUT‐059509	suke28‐6	−4.11	±0.28	40.49	±3.18
YAUT‐059511	suke28‐7	−3.58	±0.27	26.49	±3.15
YAUT‐059512	suke28‐8	−1.24	±0.47	22.95	±3.20
YAUT‐062926	suke23‐2	4.10	±0.97	21.25	±2.76
YAUT‐062928	suke23‐3	3.18	±0.47	24.25	±2.59
YAUT‐062929	suke23‐4	4.09	±0.79	27.84	±2.70
YAUT‐062931	suke23‐5	2.79	±0.91	26.82	±2.75
YAUT‐062932	suke23‐6	2.72	±0.85	20.15	±2.90
YAUT‐062933	suke23‐7	2.50	±0.91	36.37	±2.77
YAUT‐062936	suke23‐8	2.20	±0.26	27.28	±2.55
YAUT‐062937	suke23‐9	2.73	±0.61	35.76	±2.85
YAUT‐062938	suke23‐10	3.45	±0.36	32.24	±2.57
YAUT‐063902	suke25‐1	−1.65	±0.88	17.89	±2.60
YAUT‐063903	suke25‐3	−2.80	±0.62	23.49	±2.45
YAUT‐065302	suke25‐4	−1.89	±0.83	30.96	±2.45
YAUT‐065303	suke25‐5	−2.11	±1.08	4.72	±2.54
YAUT‐065304	suke25‐6	3.15	±5.56	−50.53	±6.44
YAUT‐065305	suke25‐7	4.57	±2.46	31.93	±3.69
YAUT‐065309	suke25‐09	−3.49	±2.36	−40.85	±3.38
YAUT‐058919	suke53‐1	−1.63	±0.34	−43.06	±3.05
YAUT‐058923	suke53‐2	−5.49	±1.24	−54.36	±3.53
YAUT‐058924	suke53‐3	−5.50	±0.42	−48.64	±3.06
YAUT‐058925	suke53‐4	−6.78	±1.25	−50.70	±3.55
YAUT‐058926	suke53‐5	−3.06	±1.33	−60.17	±3.60
YAUT‐059216	suke53‐6	−1.59	±1.95	−54.39	±3.60
YAUT‐059217	suke53‐7	−1.90	±2.80	−57.85	±4.43
YAUT‐059218	suke53‐8	0.14	±2.75	−63.13	±4.35
YAUT‐059219	suke53‐9	−0.88	±2.46	−57.83	±4.07
YAUT‐059224	suke56‐1	2.69	±3.74	−45.22	±5.61
YAUT‐059225	suke56‐2	−0.02	±1.17	−45.06	±3.17
YAUT‐059226	suke56‐3	−1.31	±0.69	−48.07	±2.71
YAUT‐059228	suke56‐4	−0.96	±1.38	−44.09	±3.14
YAUT‐059229	suke56‐5	−0.82	±0.91	−46.26	±2.82
YAUT‐059231	suke56‐6	−1.09	±0.89	−52.34	±2.80
YAUT‐059232	suke56‐7	−0.17	±0.74	−55.57	±2.72
YAUT‐059233	suke56‐8	−1.01	±1.42	−47.17	±3.17
YAUT‐059236	suke56‐9	0.04	±1.19	−44.93	±3.00
YAUT‐059237	suke56‐10	1.27	±1.40	−49.36	±3.14
YAUT‐059528	suke59‐1	−2.92	±1.17	−52.73	±3.42
YAUT‐059529	suke59‐2	−13.30	±1.80	−65.03	±9.98
YAUT‐059531	suke59‐3	−3.20	±0.68	−56.47	±3.13
YAUT‐059532	suke59‐4	−3.74	±0.34	−61.78	±3.43
YAUT‐059533	suke59‐5	−3.29	±0.23	−60.12	±3.00
YAUT‐059536	suke59‐6	−6.11	±0.72	−59.41	±3.16
YAUT‐059537	suke59‐7	−3.22	±0.41	−56.62	±3.04
YAUT‐059538	suke59‐8	−3.16	±0.53	−61.66	±3.07
YAUT‐059539	suke59‐9	−1.88	±0.17	−65.46	±2.97
YAUT‐059637	suke59‐10	−1.05	±0.82	−50.15	±2.45
YAUT‐062916	suke51‐1	1.30	±1.21	−58.33	±2.74
YAUT‐062917	suke51‐3	1.39	±0.48	−47.26	±2.68
YAUT‐062918	suke51‐4	2.06	±1.37	−57.48	±3.01
YAUT‐062919	suke51‐5	3.00	±1.17	−64.51	±2.71
YAUT‐062923	suke51‐6	1.85	±0.97	−63.04	±2.62
YAUT‐062924	suke51‐7	0.94	±1.89	−67.06	±3.30
YAUT‐062925	suke51‐8	3.41	±1.07	−60.42	±2.67
YAUT‐063916	suke54‐1	−2.69	±0.19	−53.42	±2.20
YAUT‐063917	suke54‐2	−2.36	±0.41	−59.01	±2.25
YAUT‐063918	suke54‐3	−3.17	±0.60	−57.98	±2.32
YAUT‐063919	suke54‐4	−2.63	±0.38	−59.95	±2.23
YAUT‐063923	suke54‐6	−1.29	±0.75	−60.31	±2.39
YAUT‐063924	suke54‐7	−3.34	±0.14	−72.18	±2.17
YAUT‐063925	suke54‐8	−2.11	±0.30	−57.79	±2.21
YAUT‐059516	suke86‐1	−2.35	±0.34	−13.18	±3.13
YAUT‐059517	suke86‐2	−2.57	±0.09	−20.04	±3.47
YAUT‐059518	suke86‐3	−3.19	±0.42	−17.74	±3.10
YAUT‐059519	suke86‐4	−2.71	±0.12	−22.47	±3.46
YAUT‐059523	suke86‐5	−2.68	±0.80	−18.31	±3.27
YAUT‐059524	suke86‐6	−4.21	±0.84	−17.18	±3.29
YAUT‐059525	suke86‐7	−4.59	±0.40	−12.61	±3.11
YAUT‐059526	suke86‐8	−5.05	±0.34	−13.43	±3.09
YAUT‐059513	suke86‐9	−3.31	±0.49	−8.69	±3.15
YAUT‐059515	suke86‐10	−3.06	±1.10	−11.32	±3.54
YAUT‐058928	suke84‐1	−5.55	±0.63	−27.20	±3.19
YAUT‐058929	suke84‐2	−5.60	±0.44	−25.56	±3.11
YAUT‐058931	suke84‐3	−4.36	±1.35	−22.42	±3.70
YAUT‐058932	suke84‐4	0.74	±1.32	−23.36	±3.67
YAUT‐058933	suke84‐5	1.97	±0.24	−21.85	±3.05
YAUT‐058936	suke84‐6	0.69	±0.71	−28.38	±3.23
YAUT‐058937	suke84‐7	−1.46	±0.85	−38.85	±3.28
YAUT‐058939	suke84‐9	−4.40	±0.56	−39.81	±3.13
YAUT‐058626	suke83‐1	−3.55	±1.55	−21.57	±2.86
YAUT‐058628	suke83‐2	−2.69	±2.27	−39.94	±3.53
YAUT‐058629	suke83‐3	−1.08	±1.88	−29.10	±3.15
YAUT‐058631	suke83‐4	0.05	±1.86	30.66	±3.29
YAUT‐058632	suke83‐5	1.04	±1.54	−12.25	±2.87
YAUT‐058633	suke83‐6	15.82	±1.13	−12.02	±2.50
YAUT‐058636	suke83‐7	1.91	±1.35	−13.64	±2.70
YAUT‐058637	suke83‐8	2.79	±1.08	−28.53	±2.45
YAUT‐058638	suke83‐9	−39.36	±1.71	−31.86	±5.41
YAUT‐058639	suke83‐10	−4.28	±1.11	−19.48	±2.51
YAUT‐062902	suke82‐1	4.07	±0.65	−40.62	±2.55
YAUT‐062903	suke82‐2	3.17	±0.73	−55.86	±2.55
YAUT‐062904	suke82‐3	2.56	±0.53	−61.16	±2.48
YAUT‐062905	suke82‐4	2.20	±0.94	−51.74	±2.63
YAUT‐062906	suke82‐5	0.72	±1.32	−33.01	±3.04
YAUT‐062909	suke82‐6	−26.81	±6.90	−45.44	±8.67
YAUT‐062911	suke82‐7	−0.41	±2.96	−31.00	±4.14
YAUT‐062912	suke82‐8	0.81	±1.61	−40.23	±3.02
YAUT‐062915	suke82‐10	−6.40	±0.55	−54.15	±2.58

*Note*: Suke29‐1 to 10, Suke26‐1 to 10, Suke28‐1 to 8, Suke23‐2 to 10, and Suke25‐1 to 10 are shown in Figure 3 as Japan‐1 to 5, respectively. Suke53‐1 to 10, Suke56‐1–10, Suke59‐1–10, Suke51‐1–7 and Suke52‐1–8 are shown in Figure 2 as Pacific‐1 to 5, respectively. Suke86‐1 to 10, Suke84‐1–9, Suke83‐1–10, and Suke82‐1–10 are shown in Figure 4 as Okhotsk‐1 to 4, respectively.

## References

[ece311288-bib-0001] Amekawa, S. , Kubota, K. , Miyairi, Y. , Seki, A. , Kawakubo, Y. , Sakai, S. , Ajithprasad, P. , Maemoku, H. , Osada, T. , & Yokoyama, Y. (2016). Fossil otoliths, from the Gulf of Kutch, Western India, as a paleo‐archive for the mid‐ to late‐Holocene environment. Quaternary International Japanese Quaternary Studies, 397, 281–288. 10.1016/j.quaint.2015.07.006

[ece311288-bib-0002] Andrews, A. H. , Kalish, J. M. , Newman, S. J. , & Johnston, J. M. (2011). Bomb radiocarbon dating of three important reef‐fish species using Indo‐Pacific Δ^14^C chronologies. Marine and Freshwater Research, 62(11), 1259–1269. 10.1071/MF11080

[ece311288-bib-0003] Aramaki, T. , Senjyu, T. , Togawa, O. , Otosaka, S. , Suzuki, T. , Kitamura, T. , Amano, H. , & Volkov, Y. N. (2007). Circulation in the Northern Japan Sea studied chiefly with radiocarbon. Radiocarbon, 49, 915–924. 10.1017/S0033822200042788

[ece311288-bib-0004] Aramaki, T. , Watanabe, S. , Kuji, T. , & Wakatsuchi, M. (2001). The Okhotsk‐Pacific seawater exchange in the viewpoint of vertical profiles of radiocarbon around the Bussol’ Strait. Geophysical Research Letters, 28, 3971–3974. 10.1029/2001GL013227

[ece311288-bib-0005] Bailey, K. M. , Quinn, T. J., II , Bentzen, R. , & Grant, W. S. (1999). Population structure and dynamics of walleye pollock, *Theragra chalcogramma* . Advances in Marine Biology, 37, 179–255.

[ece311288-bib-0006] Begg, G. A. , Friedland, K. D. , & Pearce, J. B. (1999). Stock identification and its role in stock assessment and fisheries management: An overview. Fisheries Research, 43, 1–8. 10.1016/S0165-7836(99)00062-4

[ece311288-bib-0007] Brown, R. J. , & Severin, K. P. (2009). Otolith chemistry analyses indicate that water Sr:Ca is the primary factor influencing otolith Sr:Ca for freshwater and diadromous fish but not for marine fish. Canadian Journal of Fisheries and Aquatic Sciences, 66, 1790–1808. 10.1139/F09-112

[ece311288-bib-0008] Burr, G. S. , Edwards, R. L. , Donahue, D. J. , Druffel, E. R. M. , & Taylor, F. W. (1992). Mass spectrometric ^14^C and U‐Th measurements in coral. Radiocarbon, 34, 611–618. 10.1017/S003382220006389X

[ece311288-bib-0009] Campana, S. E. (1997). Use of radiocarbon from nuclear fallout as a dated marker in the otoliths of haddock *Melanogrammus aeglefinus* . Marine Ecology Progress Series, 150, 49–56. 10.3354/meps150049

[ece311288-bib-0010] Campana, S. E. (1999). Chemistry and composition of fish otoliths: Pathways, mechanisms and applications. Marine Ecology Progress Series, 188, 263–297. 10.3354/meps188263

[ece311288-bib-0011] Campana, S. E. , Chouinard, G. A. , Hanson, J. M. , Fréchet, A. , & Brattey, J. (2000). Otolith elemental fingerprints as biological tracers of fish stocks. Fisheries Research, 46, 343–357. 10.1016/S0165-7836(00)00158-2

[ece311288-bib-0012] Campana, S. E. , Fowler, A. J. , & Jones, C. M. (1994). Otolith elemental fingerprinting for stock identification of Atlantic cod (*Gadus morhua*) using laser ablation ICPMS. Canadian Journal of Fisheries and Aquatic Sciences, 51, 1942–1950. 10.1139/f94-196

[ece311288-bib-0013] Druffel, E. R. , & Williams, P. M. (1991). Radiocarbon in seawater and organisms from the Pacific coast of Baja California. Radiocarbon, 33(3), 291–296.

[ece311288-bib-0014] Eisenmann, P. , Fry, B. , Mazumder, D. , Jacobsen, G. , Holyoake, C. S. , Coughran, D. , & Bengtson Nash, S. (2017). Radiocarbon as a novel tracer of extra‐Antarctic feeding in southern hemisphere humpback whales. Scientific Reports, 7, 4366. 10.1038/s41598-017-04698-2 28663586 PMC5491506

[ece311288-bib-0015] Galley, E. A. , Wright, P. J. , & Gibb, F. M. (2006). Combined methods of otolith shape analysis improve identification of spawning areas of Atlantic cod. ICES Journal of Marine Science, 63, 1710–1717. 10.1016/j.icesjms.2006.06.014

[ece311288-bib-0016] Gauldie, R. W. , & Nelson, D. G. A. (1990). Otolith growth in fishes. Comparative Biochemistry and Physiology. Part A, Physiology, 97, 119–135. 10.1016/0300-9629(90)90159-P

[ece311288-bib-0017] Grammer, G. L. , Fallon, S. J. , Izzo, C. , Wood, R. , & Gillanders, B. M. (2015). Investigating bomb radiocarbon transport in the southern Pacific Ocean with otolith radiocarbon. Earth and Planetary Science Letters, 424, 59–68.

[ece311288-bib-0018] Hamatsu, T. , Yabuki, K. , & Watanabe, K. (2004). Decadal changes in reproduction of walleye pollock (*Theragra chalcogramma*) off the Pacific coast of northern Japan. Fisheries Oceanography, 13, 74–83. 10.1111/j.1365-2419.2004.00311.x

[ece311288-bib-0019] Hanson, S. D. , & Stafford, C. P. (2017). Modeling otolith weight using fish age and length: Applications to age determination. Transactions of the American Fisheries Society, 146, 778–790. 10.1080/00028487.2017.1310138

[ece311288-bib-0020] Høie, H. , Otterlei, E. , & Folkvord, A. (2004). Temperature‐dependent fractionation of stable oxygen isotopes in otoliths of juvenile cod (*Gadus morhua L*.). ICES Journal of Marine Science, 61, 243–251. 10.1016/j.icesjms.2003.11.006

[ece311288-bib-0021] Honda, S. , Oshima, T. , Nishimura, A. , & Hattori, T. (2004). Movement of juvenile walleye pollock, *Theragra chalcogramma*, from a spawning ground to a nursery ground along the Pacific coast of Hokkaido, Japan. Fisheries Oceanography, 13, 84–98. 10.1111/j.1365-2419.2004.00318.x

[ece311288-bib-0022] Honkalehto, T. , McCarthy, T. , Ressler, P. , Stienessen, S. , & Jones, D. (2010). Results of the acoustic‐trawl survey of walleye pollock (*Theragra chalcogramma*) on the U.S. and Russian Bering Sea shelf in June – August 2009 (DY0909). AFSC Processed Rep. 2010‐03, 57p. Alaska Fish. Sci. Cent., NOAA, Natl. Mar. Fish. Serv., 7600 Sand Point Way NE, Seattle WA 98115.

[ece311288-bib-0023] Ishikawa, N. F. , Ogawa, N. O. , Chikaraishi, Y. , Yamaguchi, M. , Fujikura, K. , Miyairi, Y. , Yokoyama, Y. , Nagata, T. , & Ohkouchi, N. (2021). Influences of ocean currents on the diets of demersal fish communities in the Western North Pacific revealed by their muscle carbon and nitrogen isotopic compositions. Frontiers in Marine Science, 8, 8.

[ece311288-bib-0024] Itaya, K. , Miyake, H. , Wada, A. , & Miyashita, K. (2009). Distribution of walleye pollock (*Theragra chalcogramma*) larvae and juveniles off the northern Hokkaido coast from the sea of Japan to the Sea of Okhotsk. Bull. Jpn. Soc. Fish. Oceanogr. Jpn. 73.

[ece311288-bib-0025] Kalish, J. M. (1991). Effect of physiology and endolymph composition on the strontium content of bearded rock cod (*Pseudophycis barbatus*) otoliths. In S. Suga & H. Nakahara (Eds.), Mechanisms and phylogeny of mineralization in biological systems (pp. 261–265). Springer Japan. 10.1007/978-4-431-68132-8_43

[ece311288-bib-0026] Kalish, J. M. , Johnston, J. M. , Gunn, J. S. , & Clear, N. P. (1996). Use of the bomb radiocarbon chronometer to determine age of southern bluefin tuna *Thunnus maccoyii* . Marine Ecology Progress Series, 143, 1–8. 10.3354/meps143001

[ece311288-bib-0027] Kooka, K. (2012). Life‐history traits of walleye pollock, *Theragra chalcogramma*, in the northeastern Japan Sea during early to mid 1990s. Fisheries Research, 113, 35–44. 10.1016/j.fishres.2011.09.001

[ece311288-bib-0028] Kooka, K. , Takatsu, T. , Kamei, Y. , Nakatani, T. , & Takahashi, T. (1998). Vertical distribution and prey of walleye pollock in the northern Japan Sea. Fisheries Science, 64, 686–693. 10.2331/fishsci.64.686

[ece311288-bib-0029] Kubota, K. , Yokoyama, Y. , Kawakubo, Y. , Seki, A. , Sakai, S. , Ajithprasad, P. , Maemoku, H. , Osada, T. , & Bhattacharya, S. K. (2015). Migration history of an ariid Indian catfish reconstructed by otolith Sr/Ca and *δ* ^18^O micro‐analysis. Geochemical Journal, 49, 469–480. 10.2343/geochemj.2.0371

[ece311288-bib-0030] Kumamoto, Y. , Murata, A. , Kawano, T. , Watanabe, S. , & Fukasawa, M. (2013). Decadal changes in bomb‐produced radiocarbon in the Pacific Ocean from the 1990s to 2000s. Radiocarbon, 55, 1641–1650. 10.1017/S0033822200048554

[ece311288-bib-0031] Lan, H. , Hirabayashi, S. , Miyairi, Y. , & Yokoyama, Y. (2023). First dataset of dissolved inorganic radiocarbon in the Tokara Strait. Geochemical Journal, 57, 197–203. 10.2343/geochemj.GJ23018

[ece311288-bib-0032] Larsen, T. , Yokoyama, Y. , & Fernandes, R. (2018). Radiocarbon in ecology: Insights and perspectives from aquatic and terrestrial studies. Methods in Ecology and Evolution, 9, 181–190. 10.1111/2041-210X.12851

[ece311288-bib-0033] Maeda, T. , Takagi, S. , Kamei, Y. , Kajiwara, Y. , Meguro, T. , & Nakatani, T. (1993). History and methodology of walleye pollock studies. Scientific reports of Hokkaido Fisheries Experiment Station, 1–14.

[ece311288-bib-0034] McNichol, A. P. , & Aluwihare, L. I. (2007). The power of radiocarbon in biogeochemical studies of the marine carbon cycle: Insights from studies of dissolved and particulate organic carbon (DOC and POC). Chemical Reviews, 107, 443–466. 10.1021/cr050374g 17300140

[ece311288-bib-0035] Miyairi, Y. , Yokoyama, Y. , & Nagata, T. (2023). Newly designed glass apparatus to conduct stepwise dissolution experiment for radiocarbon using fish otoliths. Nuclear Instruments and Methods in Physics Research, Section B: Beam Interactions with Materials and Atoms, 539, 22–27. 10.1016/j.nimb.2023.02.031

[ece311288-bib-0036] Miyashita, K. , Tetsumura, K. , Honda, S. , Oshima, T. , Kawabe, R. , & Sasaki, K. (2004). Diel changes in vertical distribution patterns of zooplankton and walleye pollock (*Theragra chalcogramma*) off the Pacific coast of eastern Hokkaido, Japan, estimated by the volume back scattering strength (Sv) difference method. Fisheries Oceanography, 13, 99–110. 10.1111/j.1365-2419.2004.00313.x

[ece311288-bib-0037] Mori, K. , & Hiyama, Y. (2014). Stock assessment and management for walleye pollock in Japan. Fisheries Science, 80, 161–172. 10.1007/s12562-014-0720-3

[ece311288-bib-0038] Nishimura, A. , Hamatsu, T. , Yabuki, K. , & Shida, O. (2002). Recruitment fluctuation and biological responses of walleye pollock in the Pacific coast of Hokkaido. Fisheries Science, 68, 206–209. 10.2331/fishsci.68.sup1_206

[ece311288-bib-0039] Ota, K. , Yokoyama, Y. , Miyairi, Y. , Hayakawa, J. , Satoh, N. , Fukuda, H. , & Tanaka, K. (2021). Northeast Pacific seawater radiocarbon recorded in abalone shells obtained from Otsuchi Bay, Japan. Radiocarbon, 63, 1249–1258. 10.1017/RDC.2019.95

[ece311288-bib-0040] Proctor, C. H. , Thresher, R. E. , Gunn, J. S. , Mills, D. J. , Harrowfield, I. R. , & Sie, S. H. (1995). Stock structure of the southern bluefin tuna *Thunnus maccoyii*: An investigation based on probe microanalysis of otolith composition. Marine Biology, 122, 511–526. 10.1007/BF00350674

[ece311288-bib-0041] R Core Team . (2023). R: A language and environment for statistical computing. R Foundation for Statistical Computing. https://www.R‐project.org/

[ece311288-bib-0042] Rooker, J. R. , Kraus, R. T. , & Secor, D. H. (2004). Dispersive behaviors of black drum and red drum: Is otolith Sr:Ca a reliable indicator of salinity history? Estuaries, 27, 334–341. 10.1007/BF02803389

[ece311288-bib-0043] Satoh, N. (2020). Analysis of carbon dynamics in the Sanriku coastal ecosystem using radiocarbon isotopic compositions. [Doctoral dissertation]. The University of Tokyo.

[ece311288-bib-0044] Satoh, N. , Fukuda, H. , Miyairi, Y. , Yokoyama, Y. , & Nagata, T. (2019). Position‐dependent radiocarbon content of the macroalgae *Undaria pinnatifida* as an indicator of oceanographic conditions during algal growth. Journal of Oceanography, 75, 349–358. 10.1007/s10872-019-00508-7

[ece311288-bib-0045] Servettaz, A. P. , Yokoyama, Y. , Hirabayashi, S. , Kienast, M. , Miyairi, Y. , & Mohtadi, M. (2019). Dissolved inorganic radiocarbon content of the Western Coral Sea: Implications for intermediate and deep water circulation. Radiocarbon, 61, 1685–1696. 10.1017/RDC.2019.122

[ece311288-bib-0046] Shida, O. (2001). Age‐dependent bathymetric pattern of walleye pollock, *Theragra chlcogramma*, off the Pacific coast of eastern Hokkaido. Scientific Reports of Hokkaido Fisheries Experiment Station, 63, 9–20.

[ece311288-bib-0047] Solomon, C. T. , Weber, P. K. , Cech, J. J., Jr. , Ingram, B. L. , Conrad, M. E. , Machavaram, M. V. , Pogodina, A. R. , & Franklin, R. L. (2006). Experimental determination of the sources of otolith carbon and associated isotopic fractionation. Canadian Journal of Fisheries and Aquatic Sciences, 63(1), 79–89.

[ece311288-bib-0048] Springer, A. M. (1992). A review: Walleye pollock in the North Pacific – how much difference do they really make? Fisheries Oceanography, 1, 80–96. 10.1111/j.1365-2419.1992.tb00026.x

[ece311288-bib-0049] St John Glew, K. , Espinasse, B. , Hunt, B. P. , Pakhomov, E. A. , Bury, S. J. , Pinkerton, M. , Nodder, S. D. , Gutiérrez‐Rodríguez, A. , Safi, K. , Brown, J. C. , Graham, L. , Dunbar, R. B. , Mucciarone, D. A. , Magozzi, S. , Somes, C. , & Trueman, C. N. (2021). Isoscape models of the Southern Ocean: Predicting spatial and temporal variability in carbon and nitrogen isotope compositions of particulate organic matter. Global Biogeochemical Cycles, 35(9). 10.1029/2020gb006901

[ece311288-bib-0050] Sturrock, A. M. , Trueman, C. N. , Darnaude, A. M. , & Hunter, E. (2012). Can otolith elemental chemistry retrospectively track migrations in fully marine fishes? Journal of Fish Biology, 81, 766–795. 10.1111/j.1095-8649.2012.03372.x 22803735

[ece311288-bib-0061] Stuiver, M. , & Polach, H. A. (1977). Discussion Reporting of 14C Data. Radiocarbon, 19(3), 355–363. 10.1017/S0033822200003672

[ece311288-bib-0051] Sturrock, A. M. , Trueman, C. N. , Milton, J. A. , Waring, C. P. , Cooper, M. J. , & Hunter, E. (2014). Physiological influences can outweigh environmental signals in otolith microchemistry research. Marine Ecology Progress Series, 500, 245–264. 10.3354/meps10699

[ece311288-bib-0052] Toggweiler, J. R. , Druffel, E. R. M. , Key, R. M. , & Galbraith, E. D. (2019). Upwelling in the ocean basins north of the ACC: 1. On the upwelling exposed by the surface distribution of Δ^14^C. Journal of Geophysical Research, Oceans, 124, 2591–2608. 10.1029/2018JC014794

[ece311288-bib-0053] Tzadik, O. E. , Curtis, J. S. , Granneman, J. E. , Kurth, B. N. , Pusack, T. J. , Wallace, A. A. , Hollander, D. J. , Peebles, E. B. , & Stallings, C. D. (2017). Chemical archives in fishes beyond otoliths: A review on the use of other body parts as chronological recorders of microchemical constituents for expanding interpretations of environmental, ecological, and life‐history changes. Limnology and Oceanography: Methods, 15, 238–263. 10.1002/lom3.10153

[ece311288-bib-0054] Watanobe, M. (2008). Walleye pollack (suketoudara) fishery management in the Hiyama region of Hokkaido, Japan. Case Stud. Fish. Self‐Gov., 163–274.

[ece311288-bib-0055] West, J. B. , Bowen, G. J. , Cerling, T. E. , & Ehleringer, J. R. (2006). Stable isotopes as one of nature's ecological recorders. Trends in Ecology & Evolution, 21, 408–414. 10.1016/j.tree.2006.04.002 16753238

[ece311288-bib-0056] Yokouchi, K. , Amano, Y. , Ishimura, T. , & Shirai, K. (2017). Geochemical analysis of otoliths for studying the migratory ecology of fishes. Bulletin of the Japanese Society of Fisheries Oceanography, 81, 189–202. 10.34423/jsfo.81.3_189

[ece311288-bib-0057] Yokoyama, Y. , Esat, T. M. , Lambeck, K. , & Fifield, L. K. (2000). Last ice age millennial scale climate changes recorded in Huon Peninsula corals. Radiocarbon, 42, 383–401. 10.1017/S0033822200030320

[ece311288-bib-0058] Yokoyama, Y. , Miyairi, Y. , Aze, T. , Sawada, C. , Ando, Y. , Izawa, S. , Ueno, Y. , Hirabayashi, S. , Fukuyo, N. , Ota, K. , Shimizu, Y. , Zeng, Y. , Lan, H. , Tsuneoka, R. , Ando, K. , Nemoto, K. , Obrochta, S. , Behrens, B. , Tam, E. , … Nagata, T. (2022). Efficient radiocarbon measurements on marine and terrestrial samples with single stage accelerator mass spectrometry at the atmosphere and ocean research institute, University of Tokyo. Nuclear Instruments and Methods in Physics Research Section B: Beam Interactions with Materials and Atoms, 532, 62–67. 10.1016/j.nimb.2022.10.006

[ece311288-bib-0059] Yokoyama, Y. , Miyairi, Y. , Aze, T. , Yamane, M. , Sawada, C. , Ando, Y. , de Natris, M. , Hirabayashi, S. , Ishiwa, T. , Sato, N. , & Fukuyo, N. (2019). A single stage accelerator mass spectrometry at the Atmosphere and Ocean Research Institute, the University of Tokyo. Nuclear Instruments and Methods in Physics Research Section B: Beam Interactions with Materials and Atoms, 455, 311–316. 10.1016/j.nimb.2019.01.055

[ece311288-bib-0060] Yoshida, H. (1982). On the results of pollock tagging in the waters around Hokkaido. Rep. North. Jpn. Bottom Fish Conf. Fish Res Res Conf. Fisc. 1981 70–79.

